# Association between serum carotenoids levels and severe headache or migraine in adults: a cross-sectional study from NHANES

**DOI:** 10.3389/fnut.2024.1507503

**Published:** 2025-01-06

**Authors:** Tian Hu, Yufei Chen, Siyu Chen, Rui Xue

**Affiliations:** Department of Anesthesiology, Renmin Hospital, Hubei University of Medicine, Shiyan, Hubei, China

**Keywords:** migraine, carotenoid, NHANES, cross-sectional study, *α*-carotene

## Abstract

**Background:**

Carotenoids are well-established for their potent antioxidant properties; however, their potential association with severe headaches or migraines remains largely unexamined. This study was conducted to explore the relationship between serum carotenoid levels and the prevalence of severe headaches or migraines within the US population.

**Methods:**

We utilized data from the 2001–2004 National Health and Nutrition Examination Survey (NHANES), which comprised a total of 8,910 participants. Serum carotenoid levels—specifically *α*-carotene, *β*-carotene, β-cryptoxanthin, lutein/zeaxanthin, and lycopene—were quantified using high-performance liquid chromatography. Migraine status was determined based on a questionnaire. The research methodologies employed included multivariate logistic regression, subgroup analysis, and restricted cubic spline (RCS) models.

**Results:**

The prevalence of migraines in the study population was 22.37%. Multivariate logistic regression analysis indicated that serum concentrations of *α*-carotene (OR = 0.91, 95% CI: 0.85–0.97), *β*-carotene (OR = 0.88, 95% CI: 0.81–0.94), β-cryptoxanthin (OR = 0.83, 95% CI: 0.76–0.90), lutein/zeaxanthin (OR = 0.75, 95% CI: 0.67–0.85), and total carotenoids (OR = 0.79, 95% CI: 0.70–0.90) were significantly inversely correlated with severe headaches or migraines; however, no significant association was found for lycopene levels. RCS analysis showed that *β*-cryptoxanthin had an L-shaped non-linear relationship with migraine prevalence at a threshold of approximately 9.392 μg/dL, while subgroup analyses confirmed the consistent inverse association between total serum carotenoid concentrations and migraine prevalence across various groups.

**Conclusion:**

Serum concentrations of *α*-carotene, *β*-carotene, β-cryptoxanthin, lutein/zeaxanthin, and total serum carotenoids were inversely correlated with the incidence of severe headaches or migraines in US adults. This evidence indicates that carotenoids may provide a protective effect against migraines; however, further investigation is warranted to substantiate these associations and to elucidate the underlying mechanisms.

## 1 Background

Migraine is a highly prevalent and debilitating neurological disorder, affecting more than 1.5 billion people worldwide, with a higher prevalence in women (1–3). The most common symptom of a migraine is a throbbing headache that comes on repeatedly. Other symptoms include light or sound sensitivity, nausea, and vomiting (4, 5). These debilitating symptoms not only cause significant suffering for patients but also result in substantial economic burdens due to absenteeism and reduced productivity. In the U.S. alone, the direct and indirect costs associated with migraines are estimated to exceed $20 billion annually (6). While the precise pathophysiological mechanisms underlying migraines are still unknown, an increasing body of research indicates that oxidative stress is a major factor in the etiology of migraines (7, 8). Free radicals and the body’s antioxidant defenses are out of balance, which leads to oxidative stress, and can lead to cellular damage, inflammatory responses, and abnormal vasoconstriction and vasodilation, which are thought to trigger migraines (9, 10). Studies have shown that oxidative stress markers are elevated in individuals with migraines, while antioxidant levels tend to be lower (11). Therefore, nutrients with antioxidant properties, such as carotenoids, may potentially exert a protective effect by reducing the occurrence or severity of migraines.

Carotenoids are natural pigments found in plants that possess strong antioxidant and anti-inflammatory properties. Their physiological functions are primarily achieved by scavenging free radicals, reducing oxidative stress, and enhancing immune function (12, 13). Multiple research studies indicate that carotenoids have a negative correlation with the likelihood of developing chronic illnesses such as cardiovascular diseases, specific forms of cancer, and age-related macular degeneration (14–16). One example is the significant association between elevated serum levels of *β*-carotene and a decreased likelihood of developing cardiovascular disease (17). Additionally, Carotenoids like lutein and zeaxanthin have been shown to protect the eyes, particularly by defending against age-related ocular diseases (18). Although these health benefits are well-established, the specific relationship between carotenoid levels and neurological disorders, including severe headaches and migraines, remains insufficiently understood.

Our research aims to analyze the correlation between levels of serum carotenoids and the prevalence of severe headaches or migraines using data from the 2001–2004 NHANES, offering new insights into the potential role of nutritional interventions in migraine management.

## 2 Materials and methods

### 2.1 Data sources and study population

**Figure 1:**
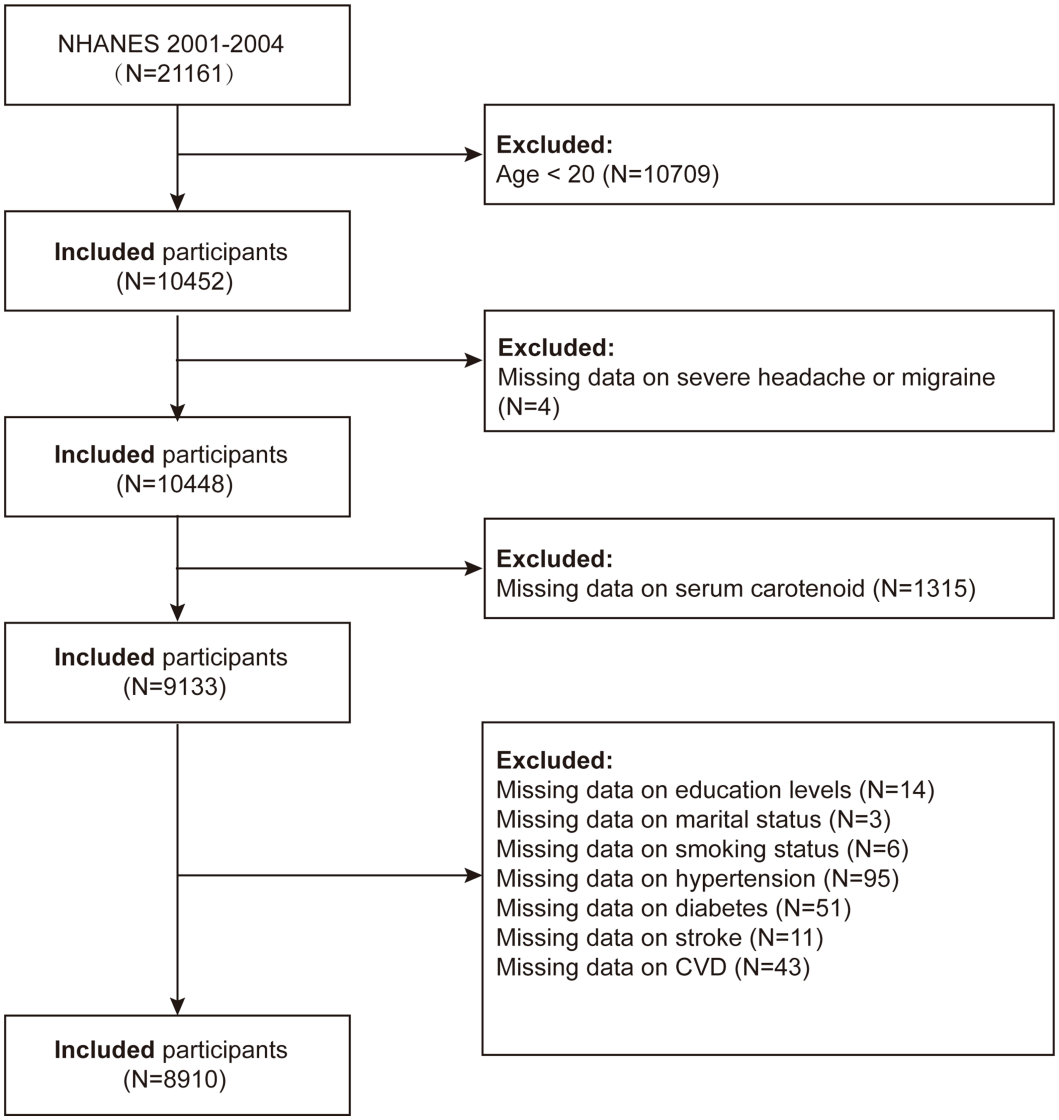
Flow chart of the study population.

NHANES is a thorough survey conducted across the entire United States by the National Center for Health Statistics (NCHS) with the goal of precisely evaluating the health and nutritional status of the American population. We used data from NHANES 2001–2004, initially including 21,161 participants in the study. The criteria for exclusion were the following: (1) participants under 20 years of age (*n* = 10,709); (2) participants missing migraine data (*n* = 4); (3) participants missing carotenoid data (*n* = 1,315); (4) participants missing educational attainment data (*n* = 14); (5) participants with incomplete marital status information (*n* = 3); (6) participants with incomplete smoking status information (*n* = 6); (7) participants missing hypertension data (*n* = 95); (8) participants missing diabetes data (*n* = 51); (9) participants missing stroke data (*n* = 11); and (10) participants missing cardiovascular disease data (*n* = 43). After applying these criteria, the final analysis included a total of 8,910 individuals (Figure 1).

### 2.2 Measurement of serum carotenoids

Fasting blood samples were obtained using red-top or royal blue-top Vacutainer^®^ tubes, frozen at −70°C, and transported with dry ice to the National Center for Environmental Health for analysis of serum carotenoid levels. High-performance liquid chromatography (HPLC) with photodiode array detection was utilized to measure serum carotenoids including *α*-carotene, *β*-carotene, β-cryptoxanthin, lycopene, and lutein/zeaxanthin. We then quantified and summed the five serum carotenoids to define total carotenoids and assess their overall effect (19, 20). National Health and Nutrition Examination Survey. Laboratory Procedure Manual: Fat Soluble Micronutrients, 2008. Available at: https://www.cdc.gov/nchs/data/nhanes/nhanes_03_04/l45vit_c_met_vitae_carotenoids.pdf.

### 2.3 Assessment of severe headache or migraine

The criteria for including migraines were based on the pain section of the NHANES self-assessment questionnaire. In the pain questionnaire (MPQ-090), individuals who answered affirmatively to the question, “Have you experienced severe headaches or migraines in the past 3 months?” were classified as migraine patients. Previous studies have validated the robustness of these findings (21–23).

### 2.4 Covariates

In this research, the variables considered were gender (male/female), age, and ethnicity (Mexican American, other Hispanic, non-Hispanic white, non-Hispanic Black, and other races). Educational achievement was categorized as less than high school, high school graduate, and more than high school. Marital status was grouped as never married, married/living with a partner, or widowed/divorced/separated. The poverty income ratio (PIR) was divided into three categories: <1.3 (low income), 1.3–3.5 (middle income), and > 3.5 (high income). Body mass index (BMI) was classified into three groups: <25 kg/m^2^, 25–30 kg/m^2^, and ≥ 30 kg/m^2^. Smoking status was identified as current smokers (≥100 cigarettes smoked and currently smoking) and never smokers (≤100 cigarettes smoked or never having smoked). A drinker was defined as an individual who had consumed at least 12 alcoholic beverages in any given year of their life. Diabetes was defined as either a doctor-diagnosed condition or a fasting blood glucose level ≥ 126 mg/dL. Hypertension was characterized by a history of diagnosis, current use of antihypertensive medication, or an average of three blood pressure readings ≥140/90 mmHg. Stroke status was recorded as “yes” or “no.” CVD encompassed a history of congestive heart failure coronary artery disease angina pectoris or myocardial infarction.

### 2.5 Statistical analyses

Statistical analyses were performed following the data analysis guidelines provided by NHANES, utilizing appropriate NHANES complex multistage sampling weights. Survey-weighted means (95% CI) were reported for continuous variables, and survey-weighted percentages (95% CI) were reported for categorical variables. Weighted linear regression or weighted chi-square tests were utilized to compare the migraine and non-migraine groups. Skewed serum carotenoid levels were transformed using a natural logarithm (ln) to approximate a normal distribution.

This study employed three weighted logistic regression models to investigate the association between serum carotenoids and severe headaches or migraines. Model 1 was unadjusted. Model 2 was adjusted for age, sex, and race/ethnicity. Model 3 was further adjusted, in addition to the covariates in Model 2, for education level, marital status, BMI, PIR, smoking, alcohol consumption, diabetes, hypertension, cardiovascular disease, and stroke. After adjusting for these covariates, the study employed restricted cubic splines (RCS) to examine the nonlinear association between serum carotenoids and migraines. Subgroup analyses and interaction tests were conducted to assess the potential confounding factors listed in the baseline table, exploring variations in the associations across subgroups. Statistical analyses were performed using R (version 4.3.3), with significance set at *p* < 0.05.

## 3 Results

### 3.1 Baseline characteristics of the study population

A study was conducted with a total of 8,910 individuals participating, with a weighted prevalence of migraine at 22.37% (20.84, 23.97%). Compared with non-migraine patients, migraine patients had significantly lower levels of *α*-carotene, *β*-carotene, β-cryptoxanthin, lutein/zeaxanthin, and total carotenoids, with respective levels of 3.26 (2.97, 3.56) μg/dL, 14.94 (13.97, 15.90) μg/dL, 7.80 (7.29, 8.32) μg/dL, 13.74 (13.20, 14.27) μg/dL, and 62.77 (60.10, 65.44) μg/dL, all showing significant differences between groups (*p* < 0.01). Additionally, the migraine group had a higher proportion of male patients, individuals with primary or high school education, those who were widowed/divorced/separated, PIR <3.5, BMI ≥30, smokers, and those with hypertension, with all showing significant differences between groups (*p* < 0.01) (Table 1).

**Table 1:** Weighted baseline characteristics of the study population.

Characteristics	Total (*n* = 8,910)	Non-migraine (*n* = 7,078)	Migraine (*n* = 1832)	*P*-value
Age (years)	45.87 (45.15,46.58)	47.10 (46.22,47.97)	41.60 (40.79,42.41)	<0.0001
Gender (%)				<0.0001
Male	47.93 (47.04,48.82)	52.06 (50.99,53.14)	33.59 (31.44,35.82)	
Female	52.07 (51.18,52.96)	47.94 (46.86,49.01)	66.41 (64.18,68.56)	
Race (%)				0.2740
Mexican American	7.24 (5.33,9.75)	7.13 (5.26,9.60)	7.61 (5.42,10.59)	
Other Hispanic	4.62 (2.95,7.17)	4.38 (2.99,6.40)	5.43 (2.70,10.62)	
Non-Hispanic White	72.83 (68.31,76.92)	73.53 (69.10,77.52)	70.40 (64.62,75.59)	
Non-Hispanic Black	10.59 (8.34,13.36)	10.24 (8.04,12.96)	11.80 (8.97,15.36)	
Other Race	4.73 (3.84,5.81)	4.72 (3.80,5.85)	4.76 (3.37,6.68)	
Education level (%)				0.0053
Less than high school	18.19 (16.71,19.78)	17.29 (15.65,19.07)	21.31 (19.15,23.65)	
High school	26.13 (24.68,27.63)	25.94 (24.27,27.67)	26.78 (24.01,29.74)	
More than high school	55.68 (53.54,57.80)	56.77 (54.23,59.27)	51.91 (48.53,55.28)	
Marital status (%)				0.3068
Never married	16.87 (14.97,18.97)	16.51 (14.56,18.67)	18.14 (15.31,21.35)	
Married/Living with partner	65.07 (63.32,66.78)	65.57 (63.59,67.49)	63.34 (60.27,66.31)	
Widowed/divorced/Separated	18.06 (16.78,19.41)	17.92 (16.53,19.40)	18.52 (16.42,20.83)	
PIR (%)				<0.0001
< 1.3	20.99 (18.82,23.33)	18.57 (16.41,20.95)	29.36 (26.21,32.71)	
1.3–3.5	35.71 (33.97,37.48)	35.31 (33.40,37.27)	37.08 (33.72,40.57)	
≥ 3.5	43.31 (40.54,46.12)	46.12 (42.94,49.32)	33.56 (30.43,36.84)	
BMI (%)				0.0002
< 25	34.17 (32.97,35.40)	34.28 (32.87,35.72)	33.80 (31.83,35.83)	
25–30	34.83 (33.22,36.47)	35.94 (34.01,37.91)	30.98 (28.36,33.73)	
≥ 30	31.00 (29.38,32.67)	29.78 (28.14,31.48)	35.22 (32.00,38.58)	
Smoking status (%)				0.2104
No	49.83 (47.63,52.02)	50.26 (48.13,52.40)	48.31 (44.65,51.98)	
Yes	50.17 (47.98,52.37)	49.74 (47.60,51.87)	51.69 (48.02,55.35)	
Alcohol intake (%)				0.0174
No	28.56 (24.81,32.63)	27.72 (24.11,31.65)	31.47 (26.50,36.91)	
Yes	71.44 (67.37,75.19)	72.28 (68.35,75.89)	68.53 (63.09,73.50)	
Hypertension (%)				0.9241
No	72.26 (70.27,74.17)	72.28 (70.30,74.18)	72.18 (69.30,74.88)	
Yes	27.74 (25.83,29.73)	27.72 (25.82,29.70)	27.82 (25.12,30.70)	
Diabetes (%)				0.0013
No	90.25 (89.30,91.12)	89.81 (88.79,90.76)	91.76 (90.47,92.89)	
Yes	9.75 (8.88,10.70)	10.19 (9.24,11.21)	8.24 (7.11,9.53)	
CVD (%)				0.3358
No	92.71 (91.53,93.73)	92.53 (91.25,93.63)	93.33 (91.50,94.79)	
Yes	7.29 (6.27,8.47)	7.47 (6.37,8.75)	6.67 (5.21,8.50)	
Stroke (%)				0.0143
No	97.50 (97.02,97.91)	97.76 (97.24,98.18)	96.63 (95.51,97.47)	
Yes	2.50 (2.09,2.98)	2.24 (1.82,2.76)	3.37 (2.53,4.49)	
α-Carotene (μg/dL)	3.68 (3.42,3.94)	3.81 (3.55,4.06)	3.26 (2.97,3.56)	<0.0001
β-Carotene (μg/dL)	16.74 (15.93,17.55)	17.26 (16.45,18.07)	14.94 (13.97,15.90)	<0.0001
β-Cryptoxanthin (μg/dL)	8.45 (8.09,8.82)	8.64 (8.28,9.01)	7.80 (7.29,8.32)	0.0004
Lutein/zeaxanthin (μg/dL)	15.13 (14.72,15.54)	15.53 (15.13,15.93)	13.74 (13.20,14.27)	<0.0001
Lycopene (μg/dL)	22.26 (21.85,22.67)	22.22 (21.79,22.65)	22.39 (21.71,23.06)	0.6239
Total carotenoids (μg/dL)	67.44 (65.42,69.46)	68.79 (66.84,70.74)	62.77 (60.10,65.44)	<0.0001

PIR, poverty income ratio; BMI, body mass index; CVD, cardiovascular disease.

### 3.2 Relationship between serum carotenoids and migraine

**Figure 2:**
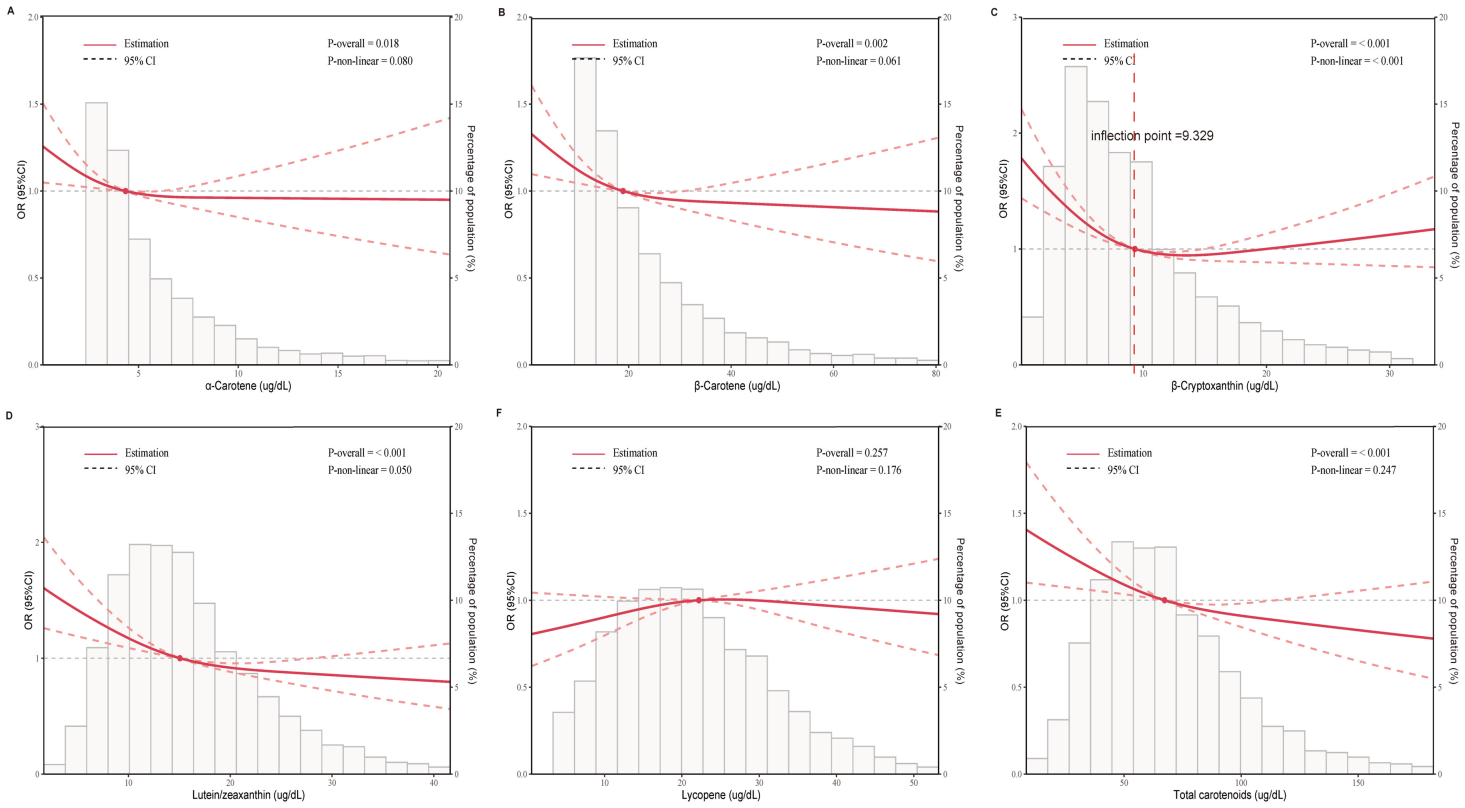
Dose-effect relationship between serum carotenoids and migraine. Age, gender, race, education level, marital status, PIR, BMI, smoking status, alcohol consumption, diabetes, hypertension, CVD, and stroke were adjusted. **(A)** *α*-Carotene and migraine; **(B)** *β*-Carotene and migraine; **(C)** β-Cryptoxanthin and migraine; **(D)** lutein/zeaxanthin and migraine; **(E)** lycopene and migraine; **(F)** total carotenoids and migraine. The solid red line represents the estimated serum carotenoid intake and the dotted line represents the 95% confidence interval. OR, odds ratio; 95% CI, 95% confidence interval.

The multivariate logistic regression analysis revealed an inverse relationship between migraine and serum carotenoids. After adjusting for all covariates, *α*-carotene (OR = 0.91, 95% CI: 0.85–0.97), *β*-carotene (OR = 0.88, 95% CI: 0.81–0.94), β-cryptoxanthin (OR = 0.83, 95% CI: 0.76–0.90), lutein/zeaxanthin (OR = 0.75, 95% CI: 0.67–0.85), and total carotenoids (OR = 0.79, 95% CI: 0.70–0.90) were significantly inversely associated with migraine. In contrast, lycopene showed no significant association with migraine (*p* > 0.05). Furthermore, when carotenoids were changed from being represented as continuous variables to categorical variables (quartiles), there was a notable decrease in the likelihood of migraine for participants in the highest quartile compared to those in the lowest quartile (Q1). (*p* for trend <0.05) (Table 2). Additionally, RCS analysis revealed no nonlinear association between *α*-carotene, *β*-carotene, lutein/zeaxanthin, total carotenoids, and migraine status (nonlinearity *p* > 0.05). Notably, β-cryptoxanthin demonstrated an L-shaped nonlinear relationship with migraine at a threshold of approximately 9.392 μg/dL (nonlinearity *p* < 0.001) (Figure 2).

**Table 2:** Multivariate logistic regression analysis between serum carotenoids and migraine.

Characteristic	Model 1		Model 2		Model 3	
	OR (95% CI)	*p*-value	OR (95% CI)	*P*-value	OR (95% CI)	*P*-value
α-Carotene						
Continuous	0.82 (0.78, 0.87)	<0.001	0.82 (0.77, 0.88)	<0.001	0.91 (0.85, 0.97)	0.005
Q1	Reference		Reference		Reference	
Q2	0.88 (0.76, 1.01)	0.061	0.88 (0.76, 1.01)	0.075	0.97 (0.84, 1.13)	0.709
Q3	0.73 (0.63, 0.84)	<0.001	0.75 (0.65, 0.88)	<0.001	0.89 (0.76, 1.04)	0.154
Q4	0.65 (0.56, 0.75)	<0.001	0.65 (0.55, 0.75)	<0.001	0.81 (0.69, 0.96)	0.015
P for trend		<0.001		<0.001		0.009
β-Carotene						
Continuous	0.79 (0.74, 0.84)	<0.001	0.80 (0.75, 0.86)	<0.001	0.88 (0.81, 0.94)	0.001
Q1	Reference		Reference		Reference	
Q2	0.81 (0.70, 0.93)	0.002	0.81 (0.70, 0.93)	0.003	0.87 (0.75, 1.00)	0.055
Q3	0.66 (0.57, 0.77)	<0.001	0.69 (0.60, 0.80)	<0.001	0.79 (0.68, 0.93)	0.004
Q4	0.63 (0.54, 0.73)	<0.001	0.66 (0.56, 0.77)	<0.001	0.78 (0.67, 0.92)	0.004
P for trend		<0.001		<0.001		0.002
β-Cryptoxanthin						
Continuous	0.83 (0.77, 0.89)	<0.001	0.76 (0.70, 0.82)	<0.001	0.83 (0.76, 0.90)	<0.001
Q1	Reference		Reference		Reference	
Q2	0.88 (0.77, 1.01)	0.080	0.81 (0.70, 0.93)	0.004	0.86 (0.74, 1.00)	0.050
Q3	0.70 (0.61, 0.81)	<0.001	0.65 (0.56, 0.75)	<0.001	0.73 (0.63, 0.85)	<0.001
Q4	0.70 (0.61, 0.81)	<0.001	0.61 (0.52, 0.71)	<0.001	0.72 (0.61, 0.85)	<0.001
P for trend		<0.001		<0.001		<0.001
Lutein/zeaxanthin						
Continuous	0.63 (0.57, 0.70)	<0.001	0.67 (0.59, 0.75)	<0.001	0.75 (0.67, 0.85)	<0.001
Q1	Reference		Reference		Reference	
Q2	0.71 (0.62, 0.81)	<0.001	0.72 (0.62, 0.83)	<0.001	0.77 (0.67, 0.89)	0.005
Q3	0.65 (0.56, 0.74)	<0.001	0.68 (0.59, 0.79)	<0.001	0.76 (0.66, 0.89)	0.005
Q4	0.54 (0.47, 0.62)	<0.001	0.58 (0.50, 0.68)	<0.001	0.67 (0.57, 0.79)	<0.001
P for trend		<0.001		<0.001		<0.001
Lycopene						
Continuous	1.18 (1.08, 1.30)	<0.001	0.97 (0.88, 1.06)	0.489	1.06 (0.96, 1.17)	0.265
Q1	Reference		Reference		Reference	
Q2	1.30 (1.12, 1.51)	0.001	1.03 (0.88, 1.20)	0.721	1.11 (0.95, 1.30)	0.192
Q3	1.33 (1.14, 1.54)	<0.001	1.01 (0.86, 1.18)	0.889	1.12 (0.96, 1.32)	0.155
Q4	1.34 (1.16, 1.56)	<0.001	1.02 (0.87, 1.19)	0.844	1.16 (0.99, 1.37)	0.070
P for trend		<0.001		0.925		0.088
Total carotenoids						
Continuous	0.73 (0.66, 0.81)	<0.001	0.68 (0.61, 0.76)	<0.001	0.79 (0.70, 0.90)	0.001
Q1	Reference		Reference		Reference	
Q2	0.84 (0.73, 0.97)	0.015	0.78 (0.67, 0.90)	0.001	0.84 (0.73, 0.98)	0.022
Q3	0.76 (0.66, 0.88)	<0.001	0.72 (0.62, 0.83)	<0.001	0.82 (0.70, 0.95)	0.010
Q4	0.65 (0.56, 0.75)	<0.001	0.62 (0.53, 0.72)	<0.001	0.75 (0.63, 0.88)	0.003
P for trend		<0.001		<0.001		<0.001

Model 1: No covariates were adjusted. Model 2: Adjusted for age, gender, and race. Model 3: Adjusted for age, gender, race, education level, marital status, PIR, BMI, smoking status, alcohol consumption, diabetes, hypertension, CVD, and stroke.

### 3.3 Subgroup analyses

**Figure 3:**
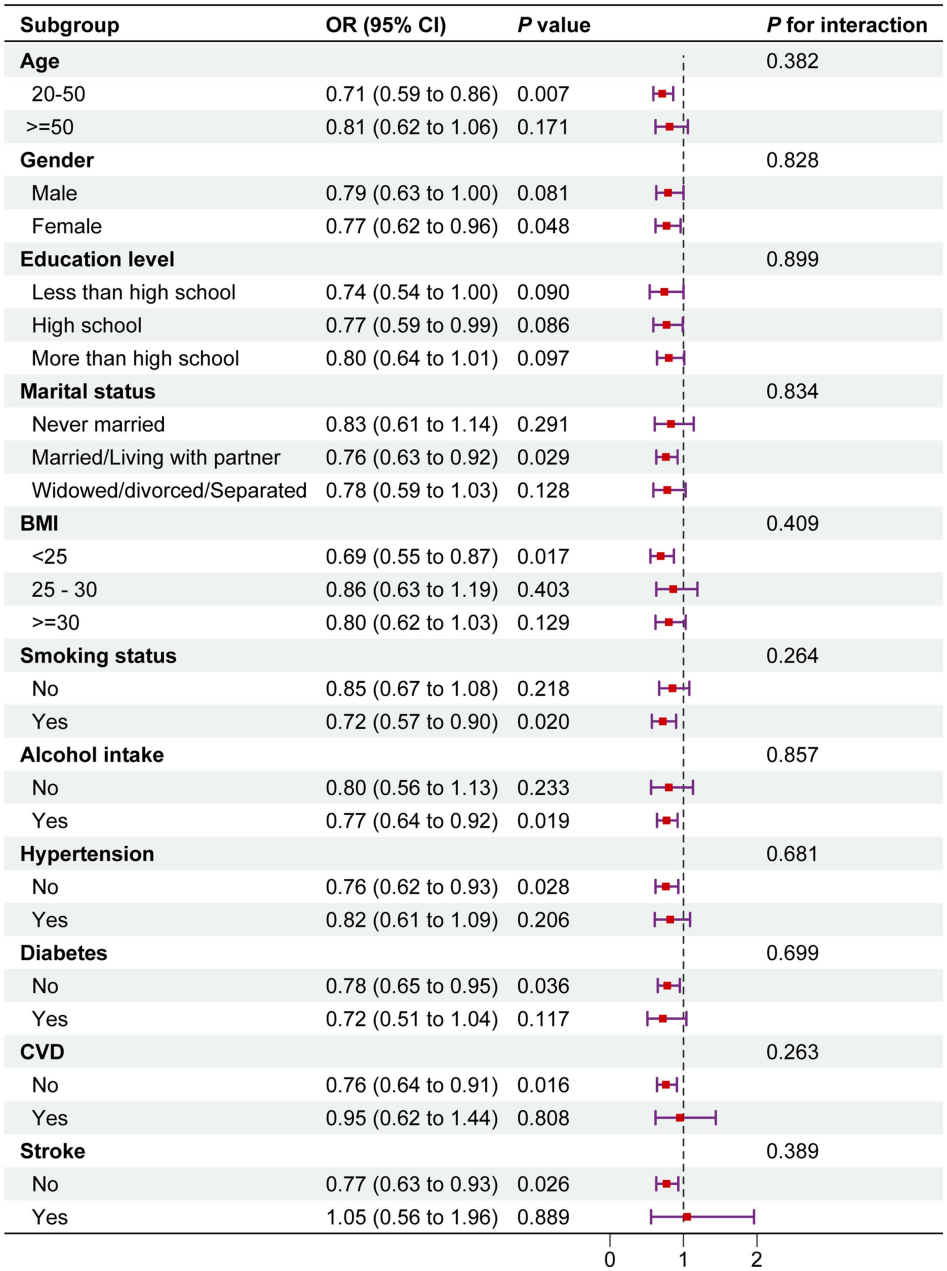
Subgroup analysis between total serum carotenoids and migraine. The above model was adjusted for gender, age, race, education level, marital status, PIR, BMI, smoking status, alcohol consumption, diabetes, hypertension, cardiovascular disease (CVD), and stroke. In each case, the model was not adjusted for the stratification variable. OR, odds ratio; CI, confidence interval.

The study results showed a negative correlation between total serum carotenoid levels and migraine prevalence across all subgroups. Moreover, across the various subgroups, there was a constant correlation between serum carotenoid levels and migraine prevalence (*P* for interaction >0.05) (Figure 3).

## 4 Discussion

The research indicated a significant inverse relationship between the prevalence of migraines and serum levels of *α*-carotene, *β*-carotene, β-cryptoxanthin, lutein/zeaxanthin, and total carotenoids. Among the carotenoids studied, β-cryptoxanthin exhibited a unique L-shaped association, suggesting that its protective effect may plateau after reaching a certain concentration. In contrast, lycopene did not show a significant association with migraines, indicating that different carotenoids may have varying effects. Subgroup analyses confirmed the robustness of the study’s findings.

This suggests that individuals with higher serum concentrations of these carotenoids are less likely to experience migraines. The protective effect of these carotenoids is primarily attributed to their strong antioxidant activity. Reactive oxygen species (ROS) production is elevated in migraines due to oxidative stress, leading to neural tissue damage and impaired vascular function, which are known contributors to the pathophysiology of this condition (24, 25). Carotenoids, as effective scavengers of ROS, may help neutralize these free radicals, thereby protecting the brain and vasculature from oxidative damage (7). Additionally, carotenoids possess anti-inflammatory properties, which may further explain their protective role in migraine prevention (26). Frequently linked to neuroinflammation, migraines are triggered by inflammatory cytokines including tumor necrosis factor-*α* and interleukin-6 (27, 28). Carotenoids, particularly lutein and *β*-carotene, have been shown to downregulate these inflammatory mediators, potentially reducing the inflammatory processes that lead to migraine attacks (29). The observed L-shaped relationship between *β*-cryptoxanthin and migraines suggests that while increasing β-cryptoxanthin levels initially provides a protective effect, this effect may reach a plateau beyond a certain point. This may indicate a threshold for the bioavailability or metabolism of carotenoids, where the body can only utilize a limited amount of β-cryptoxanthin for its antioxidant and anti-inflammatory effects. Beyond this threshold, further increases in serum levels do not provide additional protection, highlighting the need for future research to determine the optimal carotenoid levels for migraine prevention.

Studies have shown that oxidative stress markers are elevated in migraine patients, while levels of endogenous antioxidants, such as glutathione, are decreased, indicating an impaired ability to neutralize ROS in these patients. Similarly, some studies have reported that individuals whose diets are rich in carotenoids, particularly *β*-carotene and lutein, have a lower risk of neurological disorders, including migraines (30). For instance, adherence to the Mediterranean diet—which is high in fruits and vegetables that contain carotenoid—was linked to a lower incidence of migraines, according to a study by Bakırhan et al. (31). This complements the current findings, as the measurement of serum carotenoid levels provides an objective biomarker of dietary intake and antioxidant status, further supporting the protective role of these compounds in migraine prevention. The non-linear L-shaped relationship observed in this study between *β*-cryptoxanthin and migraine risk is similar to findings in other health conditions, such as cardiovascular disease. Huang et al. found a similar L-shaped association between β-cryptoxanthin levels and cardiovascular disease risk, where moderate levels provided the greatest protection, but higher levels did not offer additional benefits (17). This suggests that the protective effect of β-cryptoxanthin may be dose-dependent, with an optimal range for its beneficial effects. Results from a study in the future indicated that being exposed to numerous serum carotenoids was linked to a decreased likelihood of mortality from any cause, cardiovascular disease (CVD), and cancer. These results imply that consuming a diverse range of carotenoids in one’s diet may provide extensive health advantages (17, 19). Research by Wang et al. indicated an inverse correlation between serum carotenoid concentrations and the prevalence of CVD, with the protective effect being more pronounced for heart disease and stroke (32). Higher dietary antioxidant index scores have been associated with a lower prevalence of severe headaches or migraines (33). Additionally, Research has indicated a negative correlation between the prevalence of chronic obstructive pulmonary disease and carotenoid levels, with lutein, zeaxanthin, and *α*-cryptoxanthin being the main contributors to this inverse association (34). Research conducted by Heqing Zheng and colleagues indicated that the potential link between zinc consumption and migraines could be attributed to the antioxidant and anti-inflammatory characteristics of zinc. Furthermore, this negative association was more pronounced in participants aged 20–50 compared to those over 50 (35). In this study, there was no significant link between lycopene and migraines. This difference may be due to the way carotenoids accumulate and function in different tissues. While lycopene effectively protects against oxidative stress in the prostate and cardiovascular system, it may not have the same protective effect in the brain, where other carotenoids, like lutein and *β*-carotene, are more prevalent (36, 37).

The presence of serum carotenoids may be linked to the prevalence of migraines. Carotenoids are known for their capacity to counteract reactive oxygen species (ROS) and decrease oxidative stress, which is a significant factor in the development of migraines. Migraines are frequently connected with heightened oxidative damage, particularly in neural tissue, which is highly vulnerable to oxidative stress because of its high metabolic requirements (24, 25). By mitigating oxidative damage, carotenoids may help in preventing or reducing the frequency and severity of migraine attacks.

## 5 Strengths and limitations

The study has multiple notable strengths. To begin with, it utilizes a sizable, nationally representative sample from the NHANES dataset, enhancing the generalizability of the findings to a broader U.S. population. The utilization of NHANES ensures high-quality data collection, including serum carotenoid level measurements conducted using advanced laboratory techniques, thereby providing accurate and objective biomarkers of antioxidant status. This research emphasizes serum carotenoids rather than self-reported dietary intake, mitigating biases associated with misreporting or inaccuracies common in dietary surveys. Furthermore, this research is among the initial inquiries into the correlation between various specific carotenoids and the frequency of migraines. Previous studies have primarily concentrated on overall dietary patterns or general antioxidant intake. By examining individual carotenoids, this research offers deeper insights into how specific antioxidants may influence migraine risk. The L-shaped relationship observed between *β*-cryptoxanthin and migraine risk contributes novel information to the existing literature on antioxidants and migraines, suggesting that the carotenoids exhibit dose-dependent protective effects.

Despite these strengths, this study also has several limitations. Firstly, the cross-sectional design of the NHANES dataset constrains our ability to establish causality. This study remains uncertain whether higher carotenoid levels reduce migraine risk or if individuals with fewer migraines exhibit different dietary habits or lifestyles that contribute to elevated carotenoid levels. Longitudinal studies are necessary to more accurately assess the directionality of this relationship. Secondly, the self-reported nature of migraine diagnosis may introduce recall bias or misclassification. Finally, although this study examined multiple carotenoids, it did not investigate potential interactions between carotenoids and other nutrients, which may play synergistic roles in mitigating oxidative stress and inflammation. Future research should explore how combinations of antioxidants influence migraine risk and whether specific carotenoids are more effective when consumed alongside other nutrients.

## 6 Conclusion

The results of this research indicate that there is a negative correlation between serum levels of *α*-carotene, *β*-carotene, β-cryptoxanthin, lutein/zeaxanthin, and total carotenoids and the probability of experiencing migraines. Notably, an L-shaped relationship exists between β-cryptoxanthin and migraine risk. Increasing the intake of carotenoid-rich foods may represent a straightforward and feasible strategy for enhancing antioxidant defenses and alleviating the burden of migraines.

## Data Availability

The original contributions presented in the study are included in the article/supplementary material, further inquiries can be directed to the corresponding author.
